# The Impact of New York City’s Health Bucks Program on Electronic Benefit Transfer Spending at Farmers Markets, 2006–2009

**DOI:** 10.5888/pcd10.130113

**Published:** 2013-09-26

**Authors:** Sabrina Baronberg, Lillian Dunn, Cathy Nonas, Rachel Dannefer, Rachel Sacks

**Affiliations:** Author Affiliations: Lillian Dunn, Cathy Nonas, Rachel Dannefer, Bureau of Chronic Disease Prevention and Tobacco Control, New York City Department of Health and Mental Hygiene, New York, New York; Rachel Sacks, consultant to New York City Department of Health and Mental Hygiene, New York, New York.

## Abstract

**Introduction:**

Increasing the accessibility and affordability of fresh produce is an important strategy for municipalities combatting obesity and related health conditions. Farmers markets offer a promising venue for intervention in urban settings, and in recent years, an increasing number of programs have provided financial incentives to Supplemental Nutrition Assistance Program (SNAP) recipients. However, few studies have explored the impact of these programs on use of SNAP benefits at farmers markets.

**Methods:**

New York City’s Health Bucks Program provides SNAP recipients with a $2 coupon for every $5 spent using SNAP benefits at participating farmers markets. We analyzed approximately 4 years of electronic benefit transfer (EBT) sales data, from July 2006 through November 2009, to develop a preliminary assessment of the effect of the Health Bucks Program on EBT spending at participating markets.

**Results:**

Farmers markets that offered Health Bucks coupons to SNAP recipients averaged higher daily EBT sales than markets without the incentive ($383.07, 95% confidence interval [CI], 333.1–433.1, vs $273.97, 95% CI, 243.4–304.5, *P* < 0.001) following the introduction of a direct point-of-purchase incentive. Multivariate analysis indicated this difference remained after adjusting for the year the market was held and the neighborhood poverty level.

**Conclusion:**

When a $2 financial incentive was distributed with EBT, use of SNAP benefits increased at participating New York City farmers markets. We encourage other urban jurisdictions to consider adapting the Health Bucks Program to encourage low-income shoppers to purchase fresh produce as one potential strategy in a comprehensive approach to increasing healthful food access and affordability in low-income neighborhoods.

## Introduction

Increasing access to fresh fruits and vegetables in low-income neighborhoods and promoting consumption of these foods are important strategies for reducing the risk of heart disease, stroke, type 2 diabetes, and cancer (1–5). However, in low-income communities, limited availability and high prices present obstacles to the purchase and consumption of fresh produce (6–9). In New York City, 2009 data showed that 17.2% of residents in low-income neighborhoods reported eating no fruits and vegetables on the preceding day, compared with 8.0% of residents in high-income neighborhoods (*P* < .001) (10). Farmers markets, which are mobile and can be located throughout urban neighborhoods, offer a promising venue for intervention to decrease this disparity (11–14).

In 2005, the New York City Department of Health and Mental Hygiene (DOHMH) introduced Health Bucks, a coupon-distribution program providing financial incentives for low-income New Yorkers to shop at farmers markets in the city’s highest poverty areas. Two-dollar Health Bucks coupons were given to community-based organizations for distribution to residents for use at 11 participating markets during the annual growing season (July 1–November 15). In 2006, the DOHMH expanded Health Bucks to encourage recipients of Supplemental Nutrition Assistance Program (SNAP) benefits to use electronic benefit transfer (EBT) wireless terminals at farmers markets to purchase fruits and vegetables. EBT is the mechanism through which New York State delivers cash and SNAP benefits (formerly known as food stamps) to the state’s recipients. Funds are deposited into the accounts of individual recipients and made accessible to them via state-issued SNAP benefits cards (15). In 2006 when DOHMH expanded Health Bucks, it gave SNAP recipients at some markets a $2 coupon for every $5 in EBT credits spent. The objective of this study was to examine the program’s effect on mean EBT sales and to determine via a preliminary assessment whether Health Bucks increased EBT spending in a sample of NYC farmers markets.

## Methods

Greenmarket (http://www.grownyc.org/greenmarket), the largest outdoor urban farmers market network in the United States, manages the subset of the markets in New York City that participate in the Health Bucks program. Greenmarket has been on the forefront of national efforts to encourage SNAP spending at farmers markets and has installed EBT wireless terminals at many of the markets that are members of its network. As standard practice, Greenmarket records EBT sales at markets that accept SNAP benefits.

Greenmarket provided DOHMH with records of daily EBT sales for each individual market on each day of operation from July 2006 through November 2009. Data fields included the name and address of the market, the market date, and the total value of EBT sales in the market on that date. Altogether, Greenmarket provided EBT sales data for 1,289 market days (ie, each day of operation for each individual market) over the study period. Thirteen market days were excluded because their EBT sales were not attributed to a specific market, and an additional 15 days were excluded because records reflected EBT sales that took place at special events rather than ongoing farmers markets. Because Health Bucks are offered only during the growing season, defined as July 1 through November 15, days outside these dates were excluded (193 days). After these exclusions, our sample included EBT sales from 24 markets, operating for 45 days on average, for a total of 1,068 market days. Markets included in the final sample spanned all 5 boroughs of New York City, and 18 of the markets were located in high-poverty neighborhoods (≥20% of residents earned less than 200% of the federal poverty level [FPL]). Eleven of the 24 markets in the sample did not accept Health Bucks during the study period. Four markets had EBT sales data before and after they began accepting Health Bucks, and the remaining 9 markets had sales data available after the market began accepting Health Bucks coupons. The number of markets represented each year varied; our data included 8 markets in years 2006 and 2007, 15 in 2008, and 23 in 2009.

Independent *t* tests were used to assess differences in mean daily EBT sales among farmers markets. First, for each year of the study period, we compared differences in mean daily EBT revenue among markets that participated in the Health Bucks program and those that did not. Next, we examined a subset of markets for which EBT data were available both before and after Health Bucks coupons were offered. These markets each had a different number of market days before and after the introduction of Health Bucks because of variation in market days from year to year. Also, for one market, there was only one year of data before Health Bucks coupons were introduced compared with 3 years of data after introduction. Because sales before and after Health Bucks coupons introduction at individual markets would be correlated with each other, we used a linear mixed model to account for this dependency while assessing whether adding Health Bucks coupons as an incentive increased average daily EBT sales at these markets. Finally, we used a linear regression model to examine the impact of Health Bucks coupons on mean EBT market revenue. We adjusted for market year, because annual increases in funding of Health Bucks would be expected to cause shifts in related EBT sales each year. We also adjusted for neighborhood poverty level, because EBT use would be expected to vary with neighborhood poverty levels. FPL thresholds were used to define the neighborhood poverty level; a high-poverty neighborhood was defined as one in which 20% of residents or more earned less than 200% of FPL, a determination consistent with DOHMH standard practice. For all significance testing, the α level was set at *P* < .05. We used SPSS for Windows version 18.0 (SPSS Inc, Chicago, Illinois) to conduct all analyses.

## Results

The number of farmers markets accepting SNAP benefits via EBT terminals increased from 8 in 2006 to 23 in 2009. Average daily per-market EBT sales among all markets accepting SNAP benefits rose from $114.55 in 2006 to $465.87 in 2009. Among these markets, participation in the Health Bucks Program also expanded over time. In 2006, 5 Greenmarket markets participated in Health Bucks; this total grew to 12 participating Greenmarket markets in 2009 (Table).

**Table:** Characteristics of New York City Greenmarkets^a^ by Year, 2006–2009

Year	2006	2007	2008	2009
Number of Greenmarkets accepting EBT	8	8	15	23
Number of Greenmarkets offering Health Bucks incentives	5	6	8	12
Number of Greenmarket market days^b^ with EBT data	120	177	272	499

Abbreviation: EBT, electronic benefit transfer.

a Greenmarket (http://www.grownyc.org/greenmarket), an outdoor urban farmers market network, manages the subset of the markets in New York City that participate in the Health Bucks program.

b Includes each day of operation for each individual market.

**Figure 1:**
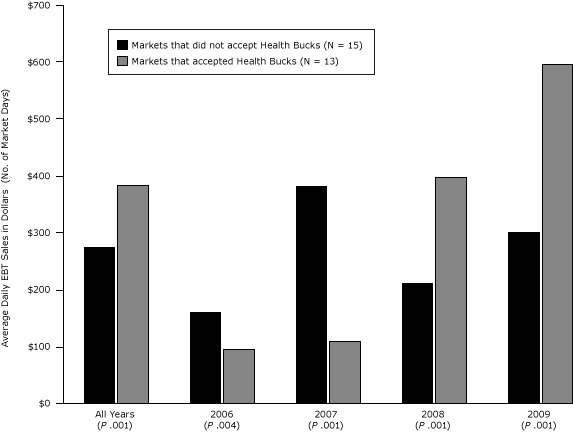
Average daily EBT sales at New York City farmers markets with and without the Health Bucks incentive, from 2006 through 2009. From 2006 through 2007, Health Bucks coupons were distributed primarily through community-based organizations. From 2008 through 2009, they were distributed primarily as an incentive for using EBT at farmers markets. Values are in dollars (followed by number of market days) and represent average sales per market day. Abbreviation: EBT, electronic benefit transfer.

During the first 2 years of the study period, markets that participated in the Health Bucks Program had lower average daily EBT sales than nonparticipating markets (Figure 1). In 2006, average daily EBT sales in participating markets were $94.64 (95% confidence interval [CI], 71.0–118.3), compared with $161.00 (95% CI, 119.2–202.8) among nonparticipating markets (*P* = .004); in 2007, participating markets averaged $109.15 (95% CI, 81.8–136.5) in daily EBT sales, compared with $381.28 (95% CI 316.4–446.2) among nonparticipating markets (*P* < .001). However, this balance shifted in the later years of analysis. In 2008, when the SNAP incentive expanded and the majority of coupon distribution shifted to EBT, participating markets documented $397.17 (95% CI, 310.5–483.8) in average daily EBT revenues, compared with $211.85 (95% CI, 161.0–262.7) at nonparticipating markets (*P* < .001); in 2009, participating markets showed nearly double the average daily EBT revenues of nonparticipating markets ($595.73, 95% CI, 495.4–696.1, vs $301.19, 95% CI, 253.9–348.4, *P* < 0.001).

**Table:** Average Daily Electronic Benefit Transfer (EBT) Sales

Year	Markets with EBT Not Accepting Health Bucks, $ (No. of Market Days)	Markets with EBT Accepting Health Bucks, $ (No. of Market Days)	*P* Value
All years	274 (396)	383 (672)	<.001
2006	161 (36)	95 (84)	.004
2007	381 (40)	109 (137)	<.001
2008	212 (100)	397 (172)	<.001
2009	301 (220)	596 (279)	<.001

To examine potential confounders, our linear regression model adjusted for the income level of the neighborhood in which the markets were located and the year in which data were collected. We found that after controlling for these factors, markets participating in Health Bucks averaged $170.79 more in daily EBT sales than nonparticipating markets (95% CI, 102.4–239.1, *P* < .001).

**Figure 2:**
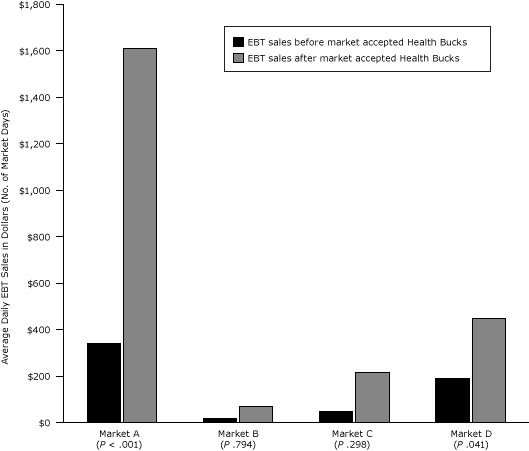
Average daily EBT sales at select New York City farmers markets before and after markets accepted Health Bucks, from 2006 through 2009. Analysis is limited to markets that accepted EBT both before and after accepting Health Bucks. Values are in dollars (followed by number of market days) and represent average sales per market day. Abbreviation: EBT, electronic benefit transfer.

To account for variation in market size, types of vendors, and operational hours that may have affected our comparison of EBT sales data across markets, we examined a subset of farmers markets for which EBT data were available both before and after Health Bucks were offered. We compared sales data within each individual market before and after the Health Bucks EBT incentive was introduced and found significant increases in EBT sales after introduction of the Health Bucks incentive in Market A ($340.67, 95% CI, 270.1–411.3 vs $1,607.88, 95% CI, 1341.30–1874.50, *P* < .001) and Market B ($18.00, 95% CI, 1.1–34.9 vs $66.53, 95% CI, 50.2–82.9, *P* = .041). Figure 2 displays average daily EBT sales for each market before and after introduction of Health Bucks.

**Table:** Average Daily Electronic Benefit Transfer (EBT) Sales

Market	EBT Sales Before Market Accepted Health Bucks, $ (No. Market Days)	EBT Sales After Market Accepted Health Bucks, $ (No. Market Days)	*P* Value
Market A	$341 (36)	$1,608 (41)	< .001
Market B	$18 (5)	$67 (57)	.794
Market C	$48 (9)	$214 (21)	.298
Market D	$190 (19)	$449 (21)	.041

## Discussion

In this preliminary study of the impact of Health Bucks on EBT spending at NYC farmer’s markets, we found that offering a direct financial incentive to SNAP recipients using EBT at urban farmers markets in 3 low-income New York City neighborhoods was associated with significant increases in EBT sales at those markets. Among markets accepting EBT in 2008 and 2009, the average daily EBT sales at participating markets were nearly double the sales of markets that did not offer the incentive. After controlling for neighborhood income level and the year in which data were collected, increases in sales figures remained significant. Additionally, we compared sales data from 4 markets for which EBT data were available before and after the Health Bucks EBT incentive was introduced and found that the introduction of Health Bucks resulted in significant increases in EBT revenue in 2 of the 4 markets.

These preliminary results suggest Health Bucks could be a useful program model for the delivery of financial incentives that encourage low-income shoppers to visit farmers markets in neighborhoods where the availability of affordable, high quality produce in the retail environment may be limited (7,9). Our findings merit further exploration, especially considering research that suggests the consumption of fruits and vegetables may be higher among low-income people and SNAP recipients who shop in farmers markets (16,17). Studies have also documented high levels of coupon use in farmers markets among low-income older adults (11) and among participants in the Special Supplemental Nutrition Program for Women, Infants, and Children (WIC) Farmers Market Nutrition Program (12–14). However, if more jurisdictions begin to provide these types of financial incentives, then developing successful strategies for distributing them will be essential. Health Bucks coupons offer a distribution model that should be further investigated for potential application in other settings seeking to encourage SNAP spending at farmers markets.

In 2008, several years after the launch of Health Bucks and midway through our study period, the New York City Human Resources Administration provided substantial financial support for the EBT incentive. This additional funding increased the number of coupons distributed from almost 20,000 in 2007 to more than 200,000 in 2008. Health Bucks was further expanded as part of the Centers for Disease Control and Prevention (CDC’s) Communities Putting Prevention to Work program (18), which funded grants to markets to hire market managers to operate the required EBT terminals. Health Bucks is now one of the largest farmers market financial programs in the United States, and our study’s findings provide evidence suggesting the effectiveness of encouraging EBT spending at farmers markets via coupons for fresh produce.

This preliminary study has limitations. First, although our findings suggest that SNAP beneficiaries spent more at markets after the Health Bucks incentive was implemented, we cannot be certain that those individuals were buying more fruits and vegetables because farmers markets sell foods and goods other than fresh produce. Second, we acknowledge that the provision of the Health Bucks incentives may have shifted SNAP spending on fruits and vegetables from other food outlets to farmers markets, rather than increasing SNAP spending on produce overall. Alternatively, SNAP recipients may have shifted their spending from farmers markets that did not offer the Health Bucks incentive to markets that did. However, a recent study examining the effects of a subsidy for fresh fruits and vegetables for postpartum women who are beneficiaries of WIC programs indicated that incentive programs did increase purchasing and consumption of fresh produce, particularly for WIC recipients who received coupons for produce at farmers markets (12). This finding leads us to suspect that Health Bucks may have had the same effect, indicating the importance of further research to explore this possibility. Third, Greenmarket’s data do not allow us to control for variations in size, vendor type, and operational hours in our analyses, and markets included in our analyses varied across years. These differences may have affected EBT sales; however, our internal comparison analysis of mean EBT sales in the same markets before and after the incentive aimed to address this limitation by comparing sales within the same market, where these factors would be relatively constant. Fourth, although the total number of days of sales data for this study was substantial (1,068), only 24 markets are represented in this study, and the sample sizes were limited, particularly for the analysis within the same markets. Further studies are needed to determine whether these are reliable effects. Finally, our data are limited to a nonrandom sample of participating Health Bucks markets that are members of the Greenmarket network and for which daily mean EBT sales data are available. 

Future studies should address these limitations by exploring in greater detail how Health Bucks affects EBT spending in farmers markets citywide. One avenue for exploration would be to determine how Greenmarket’s data capture can be improved to allow for finer analyses of market characteristics and locations that may affect EBT sales. Second, studies should be designed to assess whether increased EBT spending at farmers markets is directly linked to the purchase of fresh produce at farmers markets in low-income neighborhoods. One way to accomplish this goal would be to track how SNAP recipients spend the financial incentives they receive through Health Bucks. Third, if purchases of fresh fruits and vegetables do increase, then we must also investigate whether increased spending on fresh produce leads to greater consumption of fruits and vegetables among this low-income population; we note that a link between the purchase of fresh produce and its consumption has not yet been reliably established. Finally, by providing SNAP recipients with $2 coupons for every $5 spent via EBT, Health Bucks increased SNAP recipients’ purchasing power by 40% (calculated as 40% of $5 = $2). However, programs across the country provide varying financial incentives, including many with a dollar-per-dollar match (12–14). Given the increasingly limited funding available for these types of programs, further research should examine the incentive level required to change SNAP purchasing patterns.

Because US health care costs for obesity and its associated health consequences reach $147 billion per year (19), municipalities are seeking to identify strategies that encourage healthier behaviors, particularly among populations at greatest risk. Improving the local food environment in low-income neighborhoods is a key avenue for exploration (20–22). We urge other urban jurisdictions to consider adapting the Health Bucks program where feasible to encourage low-income shoppers to purchase fresh produce as one potential strategy in a comprehensive approach to increasing access to and affordability of healthful food in low-income neighborhoods.
